# Editorial: Language, affordance and physics in robot cognition and intelligent systems

**DOI:** 10.3389/frobt.2023.1355576

**Published:** 2024-01-09

**Authors:** Nutan Chen, Walterio W. Mayol-Cuevas, Maximilian Karl, Elie Aljalbout, Andy Zeng, Aurelio Cortese, Wolfram Burgard, Herke van Hoof

**Affiliations:** ^1^ Machine Learning Research Lab, Volkswagen Group, Munich, Germany; ^2^ School of Computer Science, University of Bristol, Bristol, United Kingdom; ^3^ Amazon, Seattle, WA, United States; ^4^ Google Deepmind, Mountain View, CA, United States; ^5^ ATR Computational Neuroscience Laboratories, Kyoto, Japan; ^6^ Department of Engineering, University of Technology Nuremberg, Nuremberg, Germany; ^7^ Informatics Institute, University of Amsterdam, Amsterdam, Netherlands

**Keywords:** robot cognition, robot language, robot affordance, physics in robotic, intelligent systems

Humans can learn new skills and recognize new objects quickly from a small number of data points. This could be attributed to our ability to generalize concepts and transfer from one task to another. For instance, humans can easily recognize if a cuboid can be sat on even if they have never seen or used it before, an ability known as affordance perception. Likewise, humans can precisely estimate the trajectory of a moving ball by perceiving and predicting physical laws. This Research Topic asks whether robots could use similarly layered cognitive systems to learn efficiently. Recent progress has been made in this area, but many unsolved problems exist for efficient robot cognition and learning.

**FIGURE 1:**
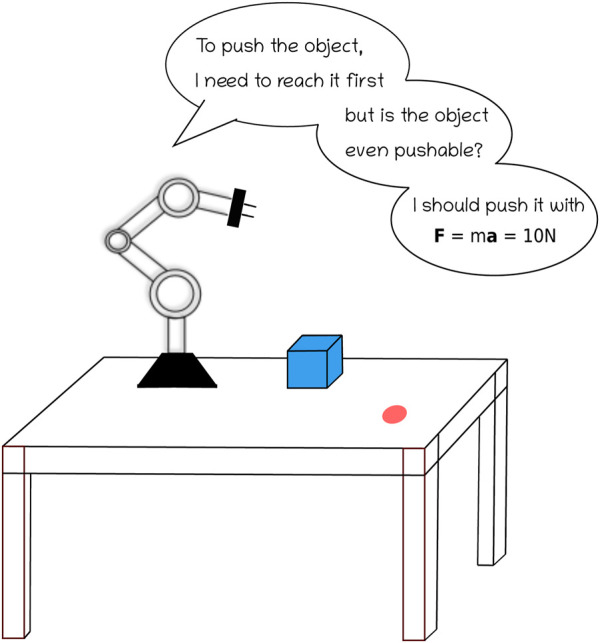
Schematic representation of a robot’s decision-making process for object manipulation as informed by natural language instructions, affordance, and physical laws.

This Research Topic discusses comprehensive updates and high-quality practices concerning machine-learning-based robot cognition. A generalist agent could strongly benefit from combining high-level affordances, intermediate-level human or robot language, and low-level prediction and recognition of physical equations (matching or learning an observed phenomenon with known physical laws) to perform in a large variety of tasks and environments (Figure 1). The goal is to improve the state of the art in language integration, affordances, and physics-based inductive biases and representations or their combination. In particular, affordances allow collecting action possibilities, enabling fast discovery and learning the environment, often from one or a few observations. In addition, natural language provides a simple and promising approach to robotic communication and cognition tasks.

On top of that, machine learning provides a common framework for jointly learning missing parameters from real-world data. Recent publications about the combination of large language models and robotics (e.g., GaTo (Reed et al., 2022), RT-1 (Brohan et al., 2022), PaLM-E (Driess et al., 2023)) showed that positive transfer is possible when additional modalities or tasks are included in the data sets. Language models can also be used to generate policy code and act as a translator between human language and robotics-related languages (Liang et al., 2023). Alternatively, physics-informed robotic learning incorporates prior knowledge and allows robots to extrapolate from a small number of samples (Lutter et al., 2019).

For this Research Topic, we invited researchers from relevant areas to engage with and discuss the challenges this topic involves. It includes six articles that provide compelling insights on language/communication and affordance.

Kartmann and Asfour introduce a framework for teaching robots spatial relationships through human interaction. This framework empowers a robot to seek human assistance for arranging objects as directed by natural language instructions, particularly when it lacks prior knowledge of the task. Additionally, the system is designed to refine its spatial awareness by learning from feedback after completing each task. Based on cylindrical distributions, this model has been shown to progressively improve learning efficiency with fewer examples, utilizing an iterative maximum likelihood estimation process for refinement. The practical application of this framework has been successfully demonstrated by a humanoid robot, confirming its potential in real-world scenarios.

Roesler presents a hybrid learning framework for grounding natural language in artificial agents, merging unsupervised and supervised techniques. This method allows agents to learn autonomously or with tutor assistance, supporting continuous learning without predefined training. The framework outperformed leading unsupervised models in accuracy and deployability, improving efficiency and accuracy when combined with supervised learning. Testing across different feedback scenarios showed the positive impact of tutor support on performance. This framework marks a step forward in enabling more intuitive communication between humans and artificial agents, essential for their use in everyday interactions.

Pourfannan et al. investigate the development of a social robot capable of speaking simultaneously in multiple languages. The study focuses on how the negative effect of background noise, particularly speech-like noise, on speech comprehension can be mitigated. It explores the impact of time expansion on speech comprehension in a multi-talker scenario through experiments involving sentence recognition, speech comprehension, and subjective evaluation tasks. The findings suggest that using time-expanded speech could make social robots more effective communicators in multilingual settings.

Deichler et al. present the generation of non-verbal communicative expressions, specifically pointing gestures, in physically situated environments for interactive agents. It emphasizes the importance of non-verbal communication modes alongside verbal communication for agents to adapt flexible communication strategies. The study presents a model that learns pointing gestures using a combination of imitation and reinforcement learning, achieving high motion naturalness and referential accuracy.

Pacheco-Ortega and Mayol-Cuevas explore a novel approach for Affordance Recognition with One-Shot Human Stances (AROS), which utilizes a one-shot learning method to identify interaction possibilities between humans and 3D scenes. Unlike traditional data-intensive methods requiring extensive training and retraining with large datasets, AROS requires only a few examples to train for new affordance instances. The system predicts affordance locations in previously unseen 3D scenes and generates corresponding 3D human bodies in those positions.

A review paper by Loeb discusses the advancement of robotic grasp affordance, delving into how robots can perceive actionable possibilities in their environments. It critiques the reliance of traditional robotics on pre-programmed data, suggesting a need for robots to learn from interaction, similar to human development. Emphasizing bio-inspired control systems, it argues for robotic middleware to emulate the adaptability of biological organisms. This could enable robots to outperform humans.

The contributions in this Research Topic collectively explored the role of language and affordances in robot cognition and intelligent systems, highlighting how their integration can lead to improved accuracy, efficiency, and flexibility. At the same time, there is untapped potential for better scaling of robot learning using physics-based representations. We believe a promising area of research will be in combining different levels of abstraction into robot decision-making pipelines. Ultimately, this combination could lead to truly generalist agents and the scaling of robot learning for multiple tasks and domains.
